# Orbital swelling as a first symptom in breast carcinoma diagnosis: a case report

**DOI:** 10.1186/1752-1947-4-211

**Published:** 2010-07-15

**Authors:** Elisa Francone, Federica Murelli, Alessandro Paroldi, Cecilia Margarino, Daniele Friedman

**Affiliations:** 1 Department of Surgery, Breast Unit, S. Martino Hospital, University of Genoa, Genoa, 16132 Italy

## Abstract

**Introduction:**

The frequency of intra-orbital metastasis in systemic cancer is a controversial topic. Of all metastatic tumors to the orbit of the eye, breast carcinoma is considered to be the most prevalent. Orbital findings typically present themselves after the diagnosis of the primary tumor, with an average delay of three to six years. In spite of that, this study reports a case in which orbital manifestation was the initial symptom in breast carcinoma diagnosis.

**Case presentation:**

A 66-year-old Italian Caucasian woman presented with a swelling located on the lower orbit of her right eye.

**Conclusions:**

Previous cases report orbital manifestations discovered secondary to breast cancer. This case demonstrates that orbital symptoms may be the primary presentation of the disease. Orbital metastasis originating from breast cancer predicts widespread metastatic disease in other organs. In the presence of an ambiguous infiltrative orbital process, diagnostic examination of the breast is recommended.

## Introduction

The most frequent sites in which breast carcinoma metastases occur are the liver, bone, lungs, skin and brain. The orbit is only associated with the spread of the disease in a small percentage of cases.

The frequency of intra-orbital metastasis in systemic cancer is a controversial topic: some authors relate an incidence of up to 30% [1]; others believe it occurs in approximately 2% to 3% of cases [2,3].

Of all metastatic tumors to the orbit - as reported in several studies [2-5] - breast carcinoma is considered to be the most prevalent primary tumor, calculated as 29% to 70% of all orbital metastases.

A recent study by the Institute of Ophthalmology and Visual Science of New Jersey Medical School argues that orbital findings typically present after diagnosis of the primary tumor, with an average delay of three to six years; although occasionally orbital metastasis may become apparent decades after the initial diagnosis of breast cancer [2].

This study reports a case from the Department of Surgery of the University of Genoa in which orbital manifestation was the initial sign in breast carcinoma diagnosis.

## Case presentation

Our patient was a 66-year-old Italian Caucasian woman with an insignificant past medical history.

She presented with a swelling located on the lower orbit of her right eye, not associated with visual disorders.

Computed tomography (CT) of the orbits revealed a thickening of her right peri-orbital soft tissues and a poor cleavage between adipose tissue, optic nerve and musculature, suggesting, on initial observation, differential diagnoses of infiltrative process (lymphoma?) and inflammatory pathology. Laboratory data only revealed an increased alkaline phosphatase (ALP) value (345 U/L).

Physical examination showed the presence of a lesion located on the external side of the left breast.

At the Breast Unit our patient underwent standard pre-operative investigations -mammography, ultrasound examination and fine needle aspiration cytology of the lesion - showing a poorly differentiated breast carcinoma.

No neoadjuvant chemotherapy treatment was suggested for our patient, who instead underwent a modified radical mastectomy and a biopsy of the peri-orbital tissue of her upper and lower eyelid.

Microscopic examination of the specimen led to the diagnosis of invasive lobular breast cancer with a massive metastatic involvement of all the 16 axillary lymph nodes, neural, vascular, and lymphatic invasion (UICC: T3/G3/N3a); in the peri-orbital tissue a widespread carcinomatous infiltration was shown by seal ring cells compatible with breast invasive lobular carcinoma derivation (UICC: T3/G3/N3a/M1).

Immunohistochemical evaluation was positive for estrogen receptor (ER: 75%) and progesterone receptor (PgR: 60%). Ki-67 proliferation index was favorably low (5%) and the oncoprotein c-erb-2 was negative (score 0).

Our patient underwent systemic oncologic evaluation (including total body CT scan and bone scintigraphy), which revealed the probable involvement of skull, first left rib, sternum-clavicular joint, dorsal rachis and bilateral iliac crests (so explaining the rise of ALP value). Blood values of carcinoembryonic antigen (CEA) rose to 20.0 μg/L and cancer antigen (CA) 15.3 to 188.00 U/mL.

Our patient received adjuvant chemotherapy (5-fluorouracil, epirubicin and cyclophosphamide (FEC) and docetaxel for three cycles) and eye radiation therapy. Because she underwent surgery fewer than five years ago, she is still under hormonal therapy (aromatase inhibitor for five years).

## Discussion

Previous cases report that orbital manifestation is usually discovered secondary to breast cancer [2,4,6-8]. This case - the first in 20 years experience of our Unit - demonstrates that orbital symptoms may be the primary presentation of the disease.

According to our findings, the agreement of histological features shows that invasive lobular carcinoma is the most common histotype in breast cancer metastatic to the orbit [5,9,10].

Orbital metastasis originating from breast cancer predicts widespread metastatic disease in other organs [2,4-6]. In our patient the systematic evaluation confirmed that metastatic spreading included several bone areas and, with reasonable suspicion, the lungs.

Our patient is monitored with annual nuclear magnetic resonance imaging of the breast, a checkwise surgical and oncological examination every six months, blood value of CEA and CA 15.3, total body and orbital examination every four months.

## Conclusions

Breast cancer presentation is not always straight forward. A patient may not detect a palpable mass.

A symptom, not strictly associated with breast cancer, brought our patient to seek medical advice. Therefore, in the presence of an ambiguous infiltrative orbital process, differential diagnosis must include breast cancer and diagnostic breast examination is highly recommended.

## List of abbreviations

ALP: alkaline phosphatase; CA 15.3: cancer antigen 15.3; CEA: carcinoembryonic antigen; CT: computed tomography; FEC: 5-fluorouracil, epirubicin and cyclophosphamide; UICC: International Union Against Cancer classification.

## Consent

Written informed consent was obtained from the patient for publication of this case report. A copy of the written consent is available for review by the Editor-in-Chief of this journal.

## Competing interests

The authors declare that they have no competing interests.

## Authors' contributions

All the authors are members of the Surgical Breast Unit and everyone has taken part in the surgical approach or in the report preparation. All authors read and approved the final manuscript.
